# Belatacept for Maintenance Immunosuppression in Lung Transplantation

**DOI:** 10.1177/2324709614546866

**Published:** 2014-09-22

**Authors:** Christine Hui, Ryan Kern, David Wojciechowski, Jasleen Kukreja, Jeffrey A. Golden, Steven R. Hays, Jonathan P. Singer

**Affiliations:** 1 UCSF Medical Center, San Francisco, CA, USA

**Keywords:** Belatacept, lung transplant, novel immunosuppression, outcomes

## Abstract

Belatacept is a novel immunosuppressant that blocks a T-cell costimulation pathway and is approved for use in adult kidney transplant recipients. Its safety and efficacy have not been established after lung transplantation. We present a case of a lung transplant recipient treated with belatacept. A 56-year-old man underwent bilateral lung retransplantation for bronchiolitis obliterans syndrome (BOS). In the third year posttransplant, he developed hemolytic uremic syndrome (HUS) attributed to tacrolimus. Tacrolimus was changed to sirolimus. One month later, he presented with worsening renal function and HUS attributed to sirolimus. Plasmapheresis and steroid pulse were initiated with clinical improvement, and sirolimus was switched to belatacept. He experienced no episodes of cellular rejection but developed recurrent BOS. Complications during treatment included anemia and recurrent pneumonias. The safety and efficacy of belatacept in lung transplantation remains unclear; further studies are needed.

## Introduction

Maintenance immunosuppression is imperative to maintain allograft function after lung transplant. While no agents are approved for use in lung transplantation, calcineurin inhibitors (CNIs) such as tacrolimus and cyclosporine are commonly used. They are, however, associated with numerous adverse effects.^1^ Mammalian target of rapamycin (mTOR) inhibitors are also used in maintenance immunosuppression in lieu or in combination with CNIs but are also associated with adverse effects.^1,2^ Thus, for lung transplant recipients intolerant of CNIs and mTOR inhibitors, options for immunosuppression are limited.

Belatacept is a novel immunosuppressant approved for use in adult kidney transplantation, but has not been studied in lung transplantation. Belatacept is a selective costimulation blocker that binds CD80 and CD86, thereby blocking CD28-mediated costimulation in the T-cell activation cascade.^3,4^ We present a case of a lung transplant recipient who developed hemolytic uremic syndrome (HUS) secondary to tacrolimus and subsequently sirolimus, who was ultimately switched to belatacept.

## Case Report

A 56-year-old male underwent bilateral lung retransplantation for bronchiolitis obliterans syndrome (BOS). Immunosuppression included tacrolimus, mycophenolate mofetil (MMF), and prednisone; azithromycin was used for BOS prophylaxis.^5^ Tacrolimus was adjusted to goal trough levels of 8 to 14 µg/L according to protocol. Surveillance bronchoscopy with transbronchial biopsies performed after transplant at months 2, 4, 6, 12, and then annually thereafter were negative for rejection.^6^ Spirometry was performed monthly for the first 6 months after transplant, at 1 year, and then annually thereafter. His peak forced expiratory volume in 1 second (FEV_1_) was 3.2 L 2 months posttransplant. Three months after transplant, he developed right bronchus intermedius (BI) stenosis requiring balloon dilation. The right BI stenosis recurred and was ultimately treated with laser ablation.

Two years after lung transplantation, the patient presented with fatigue, anemia, and progressive renal failure. Laboratory data revealed an increase in creatinine from 1.79 to 3.92 mg/dL over 6 months with an estimated glomerular filtration rate of 16 mL/min, spot protein-to-creatinine ratio 1.47, tacrolimus level 8.9 µg/L, hemoglobin 10.3 g/dL, and platelet count 224 000/µL. Urinalysis showed 2+ protein and trace blood. Urine microscopy showed hyaline and waxy casts. Renal biopsy demonstrated thrombotic microangiopathy and severe interstitial fibrosis and tubular atrophy consistent with chronic CNI nephrotoxicity. Tacrolimus was switched to sirolimus.

One month later, he presented with similar complaints. Laboratory data demonstrated a serum creatinine 4.73 mg/dL, hemoglobin 9.0 g/dL, platelet count 111 000/µL, LDH 672 U/L, fibrin D-dimer 1747 ng/mL, and a sirolimus level 14.3 µg/L. One month prior, his sirolimus level was 7.3 µg/L. A peripheral blood smear showed 3 to 4 schistocytes. ADAMTS13 activity was 55% (normal range 63% to 168%). Acute episodes of thrombocytopenic purpura can be triggered by antibodies against the von Willebrand factor resulting in decreased ADAMTS13 activity.^2^ These findings were felt to be consistent with HUS attributed to sirolimus, and so sirolimus was discontinued. The patient was treated with plasmapheresis for 4 days and intravenous (IV) methylprednisolone.^7^ The hemolysis resolved; however, the patient progressed to end-stage renal failure requiring hemodialysis.

In collaboration with hematology and renal transplantation colleagues, it was felt the risk of recurrent HUS was too great to rechallenge the patient with either calcineurin or mTOR inhibitors. Belatacept was considered as a novel option for immunosuppression. After verifying the patient’s Epstein–Barr virus (EBV) antibody serostatus, belatacept 10 mg/kg IV was initiated.^4^ MMF was resumed and prednisone maintained at 20 mg per day. He tolerated monthly belatacept dosed at 5 mg/kg without any infusion reactions for 24 months.

To assess efficacy of belatacept, transbronchial biopsies and spirometry were obtained. Biopsies were negative for rejection (A0B0) 3 and 6 months after belatacept was initiated.^6^ His FEV_1_ was 2.7 L 3 months prior to belatacept initiation. Six months after belatacept initiation, his FEV_1_ had fallen to 1.8 L. The decrease in FEV_1_ was attributed to recurrent stenosis of the right BI that required stenting. After the stent was placed, the patient required frequent readmissions for mucus plugging of the stent complicated by postobstructive pneumonias of bacterial and fungal origin. Further graft surveillance by bronchoscopy or spirometry could not be performed due to patient instability.

## Discussion

We present our center’s experience of belatacept use in a lung transplant recipient. Our patient tolerated belatacept infusions and did not have evidence of acute cellular rejection. However, due to recurrent bronchial stenoses and pulmonary infections, we could not definitively assess for chronic allograft rejection (BOS).^6^ It is unclear if his recurrent infections were influenced by belatacept. However, recurrent BI stenosis with stenting increased his risk for recurrent infections prior to belatacept initiation.

Although the efficacy of the belatacept to prevent acute cellular rejection is *suggested* by our transbronchial biopsies, we have a limited evidence base with which to make this statement. Further studies are needed to confirm this finding. It remains unknown if belatacept is an effective immunosuppressant against chronic or antibody-mediated rejection (AMR). It is also unclear whether belatacept contributed to our patient’s complications including anemia and upper respiratory tract infections or whether they were related to his comorbidities.

While belatacept has been reported in one other case in lung transplantation in abstract form only,^8^ belatacept has been widely used in kidney transplantation.^2-4,9-12^ The BENEFIT Study compared a “more intensive” or “less intensive” regimen of belatacept to cyclosporine in kidney transplant recipients. The approved dosing regimen for belatacept is based on the findings from the “less intensive” group. The study showed that belatacept had similar patient and allograft survival at 1 year when compared with cyclosporine.^3,10^ Belatacept was associated with superior renal function and a trend toward less chronic allograft nephropathy, despite a higher incidence of early acute rejection.^10^ Pestana et al found that the rate of acute cellular rejection was comparable in the “low-intensity” belatacept treatment group to cyclosporine patients by 3 years.^11^ Larsen et al also evaluated donor-specific antibodies (DSA) between treatment groups and found that DSAs were present in 3% of low-intensity belatacept group and 7% of cyclosporine group by month 24. Among kidney transplant recipients with prior acute rejection, the frequency of DSA was higher in those receiving cyclosporine (15%) compared with those receiving low-intensity belatacept (5%).^9,10^ Larsen and Pestana also found that DSAs were lower overall in those receiving belatacept; however, the incidence of AMR was not evaluated.^9,11^

Vincenti et al reported that at 1 year posttransplant the incidence of any infection was similar between low-intensity belatacept and cyclosporine.^3^ Three-year outcomes also showed that overall infection rates were similar between low-intensity belatacept and cyclosporine groups.^10,11^ The most common infections included urinary tract infections, nasopharyngitis, and cytomegalovirus (CMV) infections. Serious infection rates were similar between the 2 groups. The incidence of CMV and fungal infections was similar between belatacept and cyclosporine treatment groups.^10,11^ Although upper respiratory tract infection rates were similar between belatacept and cyclosporine groups, lung transplant recipients may be at a higher risk of infection as their transplanted allograft is continuously exposed to outside pathogens.^1,3,10^

The Food and Drug Administration (FDA) has issued warnings and precautions based on some of the findings from the BENEFIT and BENEFIT-EXT studies.^4^ Warnings and precautions with belatacept include increased risk of posttransplant lymphoproliferative disorder (PTLD), malignancy, and serious infection. Notably, clinical trials conducted in the process of seeking belatacept approval by the FDA identified EBV-negative serostatus at the time of transplantation as a strong risk factor for subsequent PTLD.^3,10,11^

In general, options for chronic immunosuppression in lung transplantation are limited. Belatacept is a first-in-class immunosuppressant that has been studied in the kidney transplant population as an alternative to CNIs. Although its safety and efficacy has been demonstrated in kidney transplant recipients, it was associated with a higher rate of graft loss and mortality in liver transplant recipients.^12^ Thus, we suggest belatacept may be cautiously considered as an option in lung transplantation for those intolerant of calcineurin or mTOR inhibitors. Further studies are urgently needed to evaluate its safety and efficacy in other solid organ transplant populations.
